# The effects of Baduanjin intervention on balance, lower limb strength, gait biomechanics and risks of fall among elderly

**DOI:** 10.1016/j.pmedr.2025.103129

**Published:** 2025-06-03

**Authors:** Shihao Xie, Chunlei Meng, Zuriyadda Sakipova, Shazlin Shaharudin

**Affiliations:** a School of Health Sciences, Universiti Sains Malaysia, Kota Bharu, Kelantan, Malaysia; b School of Sports Science, Shanxi Normal University Taiyuan Shanxi, China; c School of Pharmacy, S.D. Asfendiyarov Kazakh National Medical University, Almaty, Kazakhstan

**Keywords:** Biomechanics, Exercise intervention, Gait, Human health, Older adults

## Abstract

**Objective:**

Falls have been significantly associated with accidental deaths among individuals over 60 years. Therefore, improving balance function is critical in geriatric rehabilitation. This study explored the impacts of Baduanjin on the risk of falls in terms of balance, isometric knee joint strength, and gait parameters.

**Methods:**

Forty-two Chinese individuals were randomly divided into the Baduanjin group or walking group. Their gait, balance, lower limb strength, and fall risks were assessed at pre-, mid- and post-intervention. Any falls they experienced for the next six months were recorded. The data of this study were collected at Shanxi Normal University from September 2023 to January 2024.

**Results:**

The Baduanjin group demonstrated improved performance in the sway path balance test compared to the control group, particularly in the coronal plane (*p* < 0.05). In addition, the Baduanjin intervention enhanced the participants' gait symmetry during early and mid-stance gait phases and their maximum isometric strength of the knee extensor (*p* < 0.05).

**Conclusion:**

Baduanjin intervention is more effective in improving balance and preventing falls among the elderly than walking at the same intensity.

## Highlights

• Baduanjin intervention improved knee muscle strength among older adults.
• Baduanjin intervention improved gait symmetry and balance among older adults.
• Baduanjin intervention reduced the risk of falls among older adults.

## 1 Introduction

Falls have been linked to accidental deaths among individuals over 60 years (Li et al., 2022). Physical function declines in older adults as they age, and failure to detect incorrect body postures or restore balance increases the likelihood of falls (Thomas et al., 2019). It is impossible to maintain proper body posture and balance in activities without accurate integration of sensory input, central processing, and muscle strength development (Li et al., 2022). Therefore, it is essential to improve balance through geriatric rehabilitation.

Moderate-intensity exercise, such as Tai Chi or yoga, potentially prevents falls by increasing muscle strength and improving posture and stability when walking (Wooten et al., 2018; Joshi et al., 2024). Despite the benefits of these exercises for the elderly, the complexity of Tai Chi and yoga is difficult to learn. An alternative to these exercises is Baduanjin, a traditional Chinese Qigong treatment (Zou et al., 2017; Ye et al., 2020), which is easy to learn and cost-effective. Baduanjin offers deep meditation therapy, slow body movements, and musculoskeletal stretches that coordinate with body relaxation and mind concentration (Zou et al., 2018; Ye et al., 2019). Furthermore, studies in various clinical elderly populations have shown that Baduanjin can improve physical functions, including strength, flexibility, and balance (Liu et al., 2016; Zou et al., 2017).

Earlier studies on Baduanjin utilized subjective scales to assess fall risks (Tou et al., 2024), and did not include a control group with similar exercise intensity (Liu et al., 2020). These limitations make it challenging to determine the extent of Baduanjin's efficacy particularly in its mechanism on improving gait and balance mechanics. Therefore, the purpose of the current study was to investigate the impact of Baduanjin on the risk of falls among the elderly by assessing balance, isometric knee joint strength, and gait mechanics. We hypothesized that Baduanjin can improve balance, strength, and gait biomechanics of elderly, thereby reducing the risk of falls.

## 2 Method

The study procedures have been approved by the Human Research Ethics Committee of Universiti Sains Malaysia (USM/JEPeM/22080521) and complied with the Declaration of Helsinki. This study protocol was also registered with the International Standard Randomized Controlled Trial Number (ISRCTN15457910), on July 17, 2024. The study met the institution's guidelines for protection of human subjects concerning safety and privacy.

### 2.1 Study design

The voluntereed participants were randomly divided into the Baduanjin group or walking (control) group. Researchers who oversaw data statistical analysis were blinded to the grouping and restricted from obtaining detailed information about the group assignments.

### 2.2 Participants

A total of 46 volunteers met the inclusion criteria which were: 1) male and female; 2) age between 60 and 75 years; 3) possess normal cognitive ability and scored ≥25 in the Mini-Mental State Examination; 4) individuals who did not participate in other exercise intervention. Meanwhile, the exclusion criteria were as follows: 1) individuals who were diagnosed with diseases associated with the nervous system; 2) individuals with diabetes, cardiovascular diseases, peripheral vascular diseases, implanted electrical devices, non-ambulatory status, and systemic inflammatory arthritis; 3) individuals with vestibular dysfunction.

### 2.3 Experimental intervention

The participants in the Baduanjin group performed exercises for 12 weeks. The sessions were conducted thrice weekly for 40 min per session and monitored by the researchers. All exercise intervention programs were conducted indoors. A fitness trainer was recruited to guide the participants, and the exercises were performed according to the rhythm of selected melodies. Participants wore a heart rate monitor (H10, Polar, Finland) during each session and the average heart rate was maintained at between 65 and 70 % maximum heart rate (HR_max_). The maximum heart rate was calculated using the age formula: [220 – age] (American College of Sports Medicine, 2021). The exercise heart rate were recorded for both groups in the first and sixth weeks. The Baduanjin training protocol was established according to the recommendations of the Chinese Health-Qigong Association (*Health Qigong Management Center of General Administration of Sport of China*, 2003), comprising eight sections as described in Supplementary File 1. The training program also included a warm-up session where participants engaged in a five-minute walk on the treadmill at a self-selected speed and a cooling down session involving a full-body stretching exercise.

Participants in the control group (*n* = 23) were instructed to walk outdoors for 12 weeks, thrice weekly for 40 min per session. The participants wore a heart rate monitor during the walking sessions to ensure the exercise intensity was maintained in the same range as the Baduanjin group.

### 2.4 Measurements

The research team spent a week demonstrating and teaching the correct movements, warm-up and stretching skills for these exercises to the participants prior to the intervention program. Assessments below were performed in the first week (pre-intervention), sixth week (mid-intervention), and twelfth week (post-intervention).

#### 2.4.1 Balance function test during single-leg stance

The Mars static balance test system was used to evaluate the participant's stability (9287CA, Kistler, Switzerland), as described in a previous study (Koltermann et al., 2017). Firstly, the participants were instructed to remove their shoes before stepping onto the free-step plantar pressure test (Rogind et al., 2003). The participants were required to stand with a single-leg during the test (Fritz et al., 2015). The movement of the center of pressure was recorded during the testing posture with and without opened eyes. The sampling frequency was set to 1000 Hz, and each test was performed for 10 s.

#### 2.4.2 Isometric knee strength test

Limb dominance was defined as the preferred limb to kick a ball (Avedesian et al., 2019). Firstly, the participants were instructed to sit on the instrument used for the knee extensor muscle strength test (FCM 5530, HUR, Finland) and strapped to the seat to stabilize their torso. The knee extension angle was adjusted to 60° to test the optimal quadriceps femoris strength of the dominant limb. Subsequently, the participants were directed to exert force on the dynamometer gradually by extending their knees for approximately two seconds and maintaining their maximum strength for about three seconds. The maximum isometric muscle strength displayed on the instrument panel was recorded. Each test was conducted in triplicates at a five-minute interval.

#### 2.4.3 Gait test

Lower limb biomechanics during walking at self-selected speed was assessed in the gait lab using two high-speed cameras (GC-PX100, JVC, Japan) and Simi motion system (The ninth version, motion analysis software, Germany) and filmed at 100 Hz. The gait phase was divided into the “stance” and “swing” phases. Initially, the researcher explained and demonstrated the gait procedure on the treadmill (FDM-T, Zebris Medical, Germany) and then the participants practiced for several rounds. All participants completed seven successful trials at their self-selected speed. The self-selection speed used in this study was defined as the speed of walking during shopping (Queen et al., 2019). The trials were performed barefoot while wearing retroreflective markers placed on their acromial, anterior superior iliac spine, lateral tibia, lateral malleolus, and second metatarsal (Gimunová et al., 2020). In addition, the Simi motion analysis system and force measurement platform could not be automatically synchronized, and the frame rate could only be manually synchronized through the flash prompt.

#### 2.4.4 Morse fall scale (MFS)

The MFS (Morse et al., 1989) comprised six items: fall history, diagnosis of other diseases, use of walking aids, intravenous infusion or use of heparin sodium, gait, and cognitive status. The scale has a total score of 125 points, with a maximum of 25 points per item. A cumulative score of <25 indicates low risk, 25–44 is moderate risk, and > 45 points represent high risk (Chabot et al., 2019). Tang et al. (2010) stated that the scale has a sensitivity of 74 % and a specificity of 82 % among Chinese participants when the diagnostic threshold is 45 points. Additionally, we contacted the participants and recorded the actual number of falls every two weeks for the next six months post-intervention.

#### 2.4.5 Three-dimensional video data analysis

The video recorded by two high-speed cameras was used for three-dimensional modeling and establishing the three-dimensional coordinate system. The captured images were imported into the Corel video studio x9 software (Corel Corporation, Canada) for data synchronization. Subsequently, the Simi motion biomechanics analysis software (Simi Reality Motion Systems GmbH, Germany) was used for data analysis. The three-dimensional calibration accuracy verification function in the Simi motion analysis system was used to verify and calibrate the three-dimensional calculation points of the captured image with the peak frame calibration coordinate system before the analysis. The relative error between the three-dimensional calculated coordinates and the actual coordinates was set to ≤0.09 mm to ensure the accuracy of the results.

### 2.5 Data statistics and analysis

A *p*-value of <0.05 indicates significant statistical differences, and all data were expressed as mean ± standard deviation. The two-factor repeated measures analysis of variance (three-time points x two groups) was used to examine the main effects and interaction effects of change for each tested variable following intervention across two groups. If the main effects or interaction effects were significant, the Newman–Keuls method was applied for multiple comparisons. The statistical analysis in this study was conducted using the Statistical Package for Social Sciences version 24.0 (IBM, USA).

## 3 Results

### 3.1 Physical characteristics of participants

**Fig. 1:**
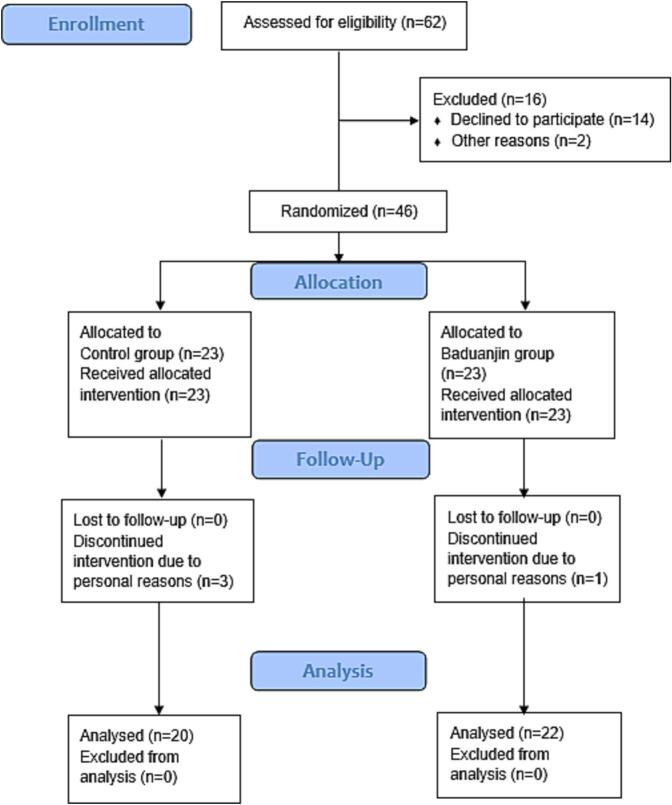
Flowchart of the 12-week exercise intervention in China from September 2023 to January 2024.

A total of 42 out of 46 recruited participants completed all exercise interventions (Baduanjin group = 22 participants; Control group = 20 participants). Four participants (Baduanjin group = three participants; Control group = one participant) dropped out of the study due to personal and family reasons. No adverse events were reported during assessment and intervention sessions in both groups. Fig. 1 presents the Consolidated Standards of Reporting Trials flow diagram of the 12-week exercise intervention.

Table 1 details the participant's physical characteristics. There was no significant difference in baseline values among the groups before intervention. The average heart rate during the first and sixth weeks of intervention for the Baduanjin group was 105.77 ± 10.32 beats/min, while the control (walking) group was 103.40 ± 8.64 beats/min [Mean differences (95 % confidence intervals) = −2.37 (−8.34, 3.59)]. The exercise intensity expressed as a percentage of maximum heart rate in the control and Baduanjin groups were 66.18 ± 5.41 % and 68.11 ± 6.61 %HR_max_, respectively.

**Table 1:** Basic information of elderly people in China in September 2023.

	Control group (*n* = 20)	Baduanjin Group (*n* = 22)	p-value
Gender	9 Male; 11 Female	12 Male; 10 Female	
Age(y)	63.7 ± 3.0	64.6 ± 2.8	0.31
Height(cm)	163.9 ± 7.6	165.5 ± 7.8	0.51
Weight(kg)	68.7 ± 7.8	69.5 ± 9.3	0.78
BMI(kg/m^2^)	25.5 ± 2.5	25.3 ± 2.6	0.74

BMI=Body mass index; Values were presented as mean (standard deviation); The p-value is tested using *t*-test.

### 3.2 Balance during single leg stance

**Fig. 2:**
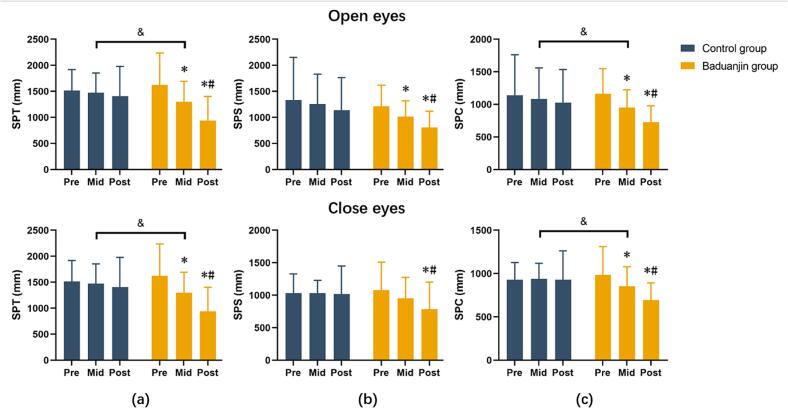
Sway path total (a), sagittal (b) and coronal plane (c) during single leg stance in elderly Chinese adults from September 2023 to January 2024. * = There was significant difference compared with the Pre; # = There was significant difference compared with the Mid; & = There was significant difference between the Baduanjin group and Control group; SPT = Sway path - total; SPS=Sway path - sagittal plane; SPC=Sway path - coronal plane. Pre, Mid, and Post represent assessments in the first week, sixth week, and twelfth week of intervention, respectively.

Fig. 2 illustrates the changes in sway path total (SPT), sagittal plane (SPS) and coronal plane (SPC) during the single-leg stance. There was no statistically significant difference between the groups before intervention for all indicators. After the intervention, the SPT [Mean differences (95 % confidence intervals) = −162.90 (−1020.48, −342.92)], SPS [Mean differences (95 % confidence intervals) = −116.99 (−536.72, −50.14)] and SPC [Mean differences (95 % confidence intervals) = −65.50 (−429.53, −157.10)] with both eyes open in Baduanjin group significantly decreased; In addition, the reduction of SPT [F (1,22) = 6.62, *p* = 0.01] and SPC [F (1,22) = 6.20, p = 0.01] in the Baduanjin group was more than that in the control group.

### 3.3 Ground reaction force during gait test

Table 2 shows the changes in ground reaction force for both groups before and after the intervention. There were no significant differences for all indicators of each group before intervention. In contrast, the early [Mean differences (95 % confidence intervals) = −0.50 (−0.73, −0.27)] and mid-stance [Mean differences (95 % confidence intervals) = −0.50 (−0.72, −0.29)] symmetry index of the Baduanjin group significantly decreased post-intervention. Meanwhile, the control group did not demonstrate statistical difference for early [Mean differences (95 % confidence intervals) = −0.17 (−0.59, 0.26)] and mid-stance [Mean differences (95 % confidence intervals) = −0.12 (−0.51, 0.28)] symmetry index after the experiment.

**Table 2:** Changes in ground reaction force before and after intervention for elderly people in China from September 2023 to January 2024.

Ground reaction force		Control group (n = 20)			Baduanjin group (n = 22)			RM-ANOVA
		Pre	Mid	Post	Pre	Mid	Post	(p-value: Time; Interaction; Group)
Left (Newton)	Heel strike	60.2 ± 17.0	60.5 ± 16.8	59.1 ± 18.6	57.7 ± 14.1	57.2 ± 12.9	56.6 ± 12.7	0.62; 0.99; 0.57
	Early stance	685.3 ± 182.2	688.3 ± 184.3	674.6 ± 201.6	673.3 ± 196.5	679.5 ± 183.9	676.4 ± 179.4	0.86; 0.76; 0.92
	Mid stance	618.3 ± 163.4	616.1 ± 171.0	599.0 ± 182.3	653.9 ± 193.0	655.3 ± 181.5	648.6 ± 189.7	0.53; 0.72; 0.42
	Toe off	55.9 ± 15.1	55.6 ± 14.5	54.2 ± 16.1	53.1 ± 14.3	54.5 ± 13.5	55.0 ± 13.9	0.96; 0.34; 0.81
Right (Newton)	Heel strike	60.4 ± 17.1	60.8 ± 16.6	60.2 ± 19.2	56.9 ± 13.5	57.3 ± 11.5	56.2 ± 13.1	0.85; 0.91; 0.38
	Early stance	690.9 ± 174.7	681.4 ± 178.3	665.0 ± 175.1	665.7 ± 184.8	669.1 ± 162.8	671.1 ± 175.4	0.59; 0.42; 0.85
	Mid stance	589.6 ± 145.0	604.1 ± 143.8	584.5 ± 164.8	655.2 ± 211.8	645.9 ± 185.2	641.6 ± 185.3	0.68; 0.82; 0.24
	Toe off	54.4 ± 13.8	55.6 ± 13.5	55.0 ± 16.2	54.9 ± 15.0	54.4 ± 12.7	54.1 ± 12.9	0.97; 0.72; 0.96
Symmetry index	Heel strike	0.7 ± 0.4	0.7 ± 0.5	0.7 ± 0.5	0.6 ± 0.5	0.6 ± 0.6	0.6 ± 0.3	0.90; 0.46; 0.36
	Early stance	1.0 ± 0.6	0.8 ± 0.6	0.8 ± 0.6	1.0 ± 0.6	0.7 ± 0.5	0.5 ± 0.3⁎	0.01; 0.14; 0.14
	Mid stance	0.9 ± 0.6	0.9 ± 0.6	0.8 ± 0.5	0.9 ± 0.5	0.7 ± 0.6	0.4 ± 0.3⁎	0.01; 0.07; 0.25
	Toe off	0.6 ± 0.4	0.7 ± 0.6	0.7 ± 0.5	0.6 ± 0.7	0.5 ± 0.5	0.6 ± 0.3	0.56; 0.59; 0.86

⁎ =There was significant difference compared with the pre-intervention level; The p-value was analyzed using repeated measures analysis of variance (RM-ANOVA). Pre, Mid, and Post represent assessments in the first week, sixth week, and twelfth week of intervention, respectively.

### 3.4 Maximum isometric knee extensor strength

**Fig. 3:**
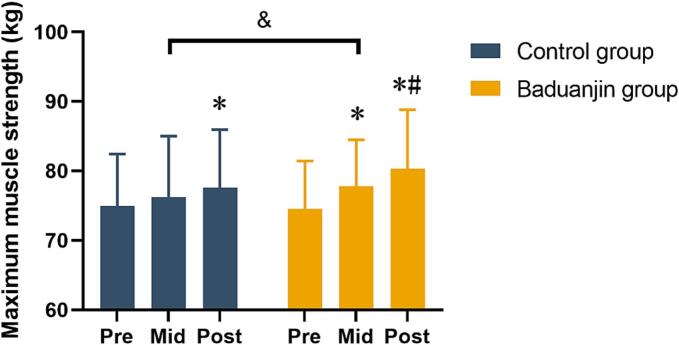
Maximum strength of knee extensor muscle in both groups before and after intervention in elderly Chinese adults from September 2023 to January 2024. * = There was significant difference compared with the Pre; # = There was significant difference compared with the Mid; & = There was significant difference between the Baduanjin group and Control group. Pre, Mid, and Post represent assessments in the first week, sixth week, and twelfth week of intervention, respectively.

There was no statistically significant difference between the groups before intervention in maximum isometric knee extensor strength (Fig. 3), but both the Baduanjin group [Mean differences (95 % confidence intervals) = 5.79 (3.78, 7.79)] and control group [Mean differences (95 % confidence intervals) = 2.65 (0.64, 4.65)] showed increment after intervention. The repeated measures analysis of variance results exhibited greater improvements in maximum isometric knee extensor strength of the Baduanjin group than the control group, indicating a significant interaction [F (1,22) = 3.63, *p* = 0.03].

### 3.5 Risk of falls

Table 3 demonstrates the MFS scores and risk of fall stratification before and after the intervention. A lower MFS score indicates a lower risk of falling. There was no statistically significant difference in the risk of falls at the baseline for both groups before intervention. After 12 weeks of intervention, the risk of falls in the Baduanjin group significantly decreased, while there were no significant changes in the control group. Meanwhile, only one elderly person (62-year-old female) in the control (i.e., walking) group experienced a fall within the six-month follow-up period.

**Table 3:** Changes in the Morse Fall Scale means and risk stratification before and after intervention for elderly people in China from September 2023 to January 2024.

	Control group (n = 20)			Baduanjin group (n = 22)			RM-ANOVA
	Pre	Mid	Post	Pre	Mid	Post	(p-value: Time; Interaction; Group)
Risk of falls	14.2 ± 10.0	13.5 ± 9.6	12.5 ± 8.1	12.7 ± 10.8	11.5 ± 10.8	9.0 ± 10.5*	0.01; 0.35; 0.46
Low risk	14 (70.0 %)	14 (70.0 %)	16 (80.0 %)	17 (77.3 %)	18 (81.8 %)	19 (86.3 %)	
Medium risk	6 (30.0 %)	6 (30.0 %)	4 (20.0 %)	5 (22.7 %)	4 (18.2 %)	3 (13.7 %)	

The p-value was analyzed using repeated measures analysis of variance (RM-ANOVA). Morse Fall Scale <25 indicates low risk, 25–44 indicates moderate risk, and > 45 indicates high risk. Pre, Mid, and Post represent assessments in the first week, sixth week, and twelfth week of intervention, respectively.

## 4 Discussion

The Baduanjin intervention in the present study improved the participant's overall and coronal sway path when standing on a single leg with eyes opened (Fig. 2). Nevertheless, there was no significant difference between the groups during single leg stance with eyes opened and closed. This discrepancy potentially resulted from the high daily demand for balance in a single-leg position such as walking. The current findings were also consistent with an earlier study, where 28 elderly individuals with an average age of 65.11 ± 6.57 years underwent 12 weeks of Baduanjin training (Ye et al., 2020). The training was performed thrice weekly and 40 min per session. The results showed that the Baduanjin exercise significantly increased the swing area during the eyes opened test and the swing path and area in the eyes closed test (Ye et al., 2020), which supported the current study findings.

The ability to maintain a stable one-legged standing posture for a fixed period is crucial for older adults. They strategically reduce walking speed to adapt to different environmental conditions, requiring longer single-limb support in the long term (Bruyneel et al., 2023). Improving the sway path during eyes closed helps minimize dependence on visual feedback to maintain balance (Low et al., 2017). This improvement is essential for those with poorer vision, a common occurrence among the elderly. A greater degree of visual field loss will increase the sway speed and center of pressure area when standing. This impairment is mainly reflected in the changes in the coronal plane, while the stability of the sagittal position is maintained through the supplement of vestibular input. Vestibular input can promote the activities of the soleus and tibialis anterior muscles, resulting in corresponding ankle torque changes in response to sagittal postural sway (Fitzpatrick et al., 1994).

Improvements in balance and gait symmetry index in the Baduanjin group in the present study (Table 2) may be due to the increased strength and stability of the participant's lower limbs and torso. Sections one, three, four and five of the Baduanjin require the participants to continuously stretch and twist their torso while maintaining physical stability, which substantially improves the core strength and stability of the torso. Meanwhile, sections two, seven, and eight involved squatting and using the participant's body weight to increase the load of the leg. These activities put the leg muscles to work, thus increasing lower limb strength and stability. Walking ability and reduced risk of falls in the elderly are closely related to knee strength (Cebolla et al., 2015). According to reports, the decline in gait performance of older adults is caused by weakened muscles in the lower limb (Correa-de-Araujo et al., 2017; Muehlbauer et al., 2018). The strength of the muscles directly affects whether the body can ultimately maintain balance (Leroux et al., 2006). Thus, the Baduanjin training improved balance and gait symmetry, which may be beneficial for reducing the risk of falling among older adults.

Improving lower limb strength is critical in preventing falls among middle-aged and elderly individuals. Lower limb strength significantly impacts walking speed and stability and is closely related to daily falls among older adults (Ye et al., 2022). In the current study, the knee extensor muscle strength was evaluated at a joint angle of 60° using an isometric muscle strength tester. The results demonstrated that 12-week interventions could improve the strength of knee joint muscles in elderly individuals (Fig. 3). Moreover, the Baduanjin group exhibited highly improved muscle strength compared to the walking group. Similarly, a study by Ye et al.,(2022) also found that the Baduanjin intervention significantly improved the participants' lower limb strength in the quadriceps and hamstring muscle strength test after 24 weeks. Therefore, the Baduanjin and walking interventions in the current study were effective in improving the knee isometric strength of the elderly.

The MFS is valuable in assessing fall risk among elderly patients, allowing researchers to dynamically evaluate their condition and modify activities to reduce their fall rates effectively. Furthermore, this scale has shown high validity (0.98) and reliability (0.97) in a study among the Chinese population (Chow et al., 2007). In the present study, the Baduanjin intervention reduced the fall risk score among the elderly, while the walking intervention (control group) showed no changes from the baseline. After 12 weeks, the moderate-risk population in the Baduanjin group decreased from 22.7 % to 13.7 %, indicating improvements in the risk classification of some participants. The current study revealed that Baduanjin can improve the fall risk of the elderly through increased balance, lower limb strength, and gait symmetry.

There are several limitations of this study. Firstly, this study focused on the elderly population without comorbidities which limit the generalization of the results. Secondly, this study did not set up a control group comprising healthy older adults without any intervention as it may not be ethically correct to let elderly without any physical activity. Finally, the testing indicators of this study were not exhaustive, as the physiological parameters were not evaluated.

It is recommended that future research expand the sample size to include elderly patients with different diseases, particularly those with skeletal muscle system conditions and metabolic diseases. Future research can also enrich the testing parameters by monitoring glucose and lipid metabolism levels and subsequent fall rates for several months or years. Additionally, further exploration can be conducted on the mechanism by which Baduanjin improves the physical function of older adults.

## 5 Conclusion

Baduanjin intervention significantly improved knee muscle strength and balance function among older adults compared to the control group. In addition, Baduanjin exercise improved gait symmetry better than the control group. Finally, Baduanjin significantly reduced the MFS score and risk of falls among the elderly population.In summary, Baduanjin intervention is superior in improving function and preventing falls among the elderly compared to walking intervention with the same intensity.

## CRediT authorship contribution statement

**Shihao Xie:** Writing – review & editing, Writing – original draft, Visualization, Project administration, Investigation, Formal analysis, Data curation. **Chunlei Meng:** Writing – review & editing, Supervision, Resources, Project administration, Methodology. **Zuriyadda Sakipova:** Writing – review & editing, Validation, Project administration, Methodology. **Shazlin Shaharudin:** Writing – review & editing, Supervision, Resources, Project administration, Methodology, Funding acquisition, Conceptualization.

## Funding statement

This work was supported by the 10.13039/501100002385Ministry of Higher Education Malaysia under the Fundamental Research Grant Scheme [Project No: FRGS/1/2024/SKK05/USM/02/2].

## Declaration of competing interest

The authors declare that they have no known competing financial interests or personal relationships that could have appeared to influence the work reported in this paper.

## Data Availability

Data will be made available on request.

## References

[bb0005] American College of Sports Medicine (2021).

[bb0010] Avedesian J.M., Judge L.W., Wang H., Dickin D.C. (2019). Kinetic analysis of unilateral landings in female volleyball players after a dynamic and combined dynamic-static warm-up. J. Strength Cond. Res..

[bb0015] Bruyneel A.-V., Reinmann A., Gafner S.C., Sandoz J.-D., Duclos N.C. (2023). Does texting while walking affect spatiotemporal gait parameters in healthy adults, older people, and persons with motor or cognitive disorders? A systematic review and meta-analysis. Gait Posture.

[bb0020] Cebolla E.C., Rodacki A.L.F., Bento P.C.B. (2015). Balance, gait, functionality and strength: comparison between elderly fallers and non-fallers. Braz. J. Phys. Ther..

[bb0025] Chabot J., Beauchet O., Fung S., Peretz I. (2019). Decreased risk of falls in patients attending music sessions on an acute geriatric ward: results from a retrospective cohort study. BMC Complement. Altern. Med..

[bb0030] Chow S.K.Y., Lai C.K.Y., Wong T.K.S., Suen L.K.P., Kong S.K.F., Chan C.K., Wong I.Y.C. (2007). Evaluation of the Morse fall scale: applicability in Chinese hospital populations. Int. J. Nurs. Stud..

[bb0035] Correa-de-Araujo R., Harris-Love M.O., Miljkovic I., Fragala M.S., Anthony B.W., Manini T.M. (2017). The need for standardized assessment of muscle quality in skeletal muscle function deficit and other aging-related muscle dysfunctions: a symposium report. Front. Physiol..

[bb0040] Fitzpatrick R., Burke D., Gandevia S.C. (1994). Task-dependent reflex responses and movement illusions evoked by galvanic vestibular stimulation in standing humans. J. Physiol..

[bb0045] Fritz N.E., Marasigan R.E.R., Calabresi P.A., Newsome S.D., Zackowski K.M. (2015). The impact of dynamic balance measures on walking performance in multiple sclerosis. Neurorehabil. Neural Repair.

[bb0050] Gimunová M., Zvonař M., Sebera M., Turčínek P., Kolářová K. (2020). Special footwear designed for pregnant women and its effect on kinematic gait parameters during pregnancy and postpartum period. PLoS One.

[bb0055] (2003). Health Qigong Management Center of General Administration of Sport of China.

[bb0060] Joshi R., Kulkarni N., Kulkarni C.A., Bansal P. (2024). Impact of tai chi and Aerobic exercise on cognitive function, balance, cardiovascular fitness, and quality of life in older adults: randomized control trial. Cureus.

[bb0065] Koltermann J., Gerber M., Beck H., Beck M. (2017). Validation of the HUMAC balance system in comparison with conventional force plates. Technologies.

[bb0070] Leroux A., Pinet H., Nadeau S. (2006). Task-oriented intervention in chronic stroke: changes in clinical and laboratory measures of balance and mobility. Am. J. Phys. Med. Rehabil..

[bb0075] Li H., Qiu X., Yang Z., Zhang Z., Wang G., Kim Y., Kim S. (2022). Effects of cha-cha dance training on the balance ability of the healthy elderly. Int. J. Environ. Res. Public Health.

[bb0080] Liu X., Gao J., Yin B., Yang X., Bai D. (2016). Efficacy of Ba Duan Jin in improving balance: a study in Chinese community-dwelling older adults. J. Gerontol. Nurs..

[bb0085] Liu X., Seah J.W.T., Pang B.W.J., Tsao M.A., Gu F., Ng W.C., Tay J.Y.R., Ng T.P., Wee S.L. (2020). A single-arm feasibility study of community-delivered Baduanjin (qigong practice of the eight brocades) training for frail older adults. Pilot Feasibility Studies.

[bb0090] Low D.C., Walsh G.S., Arkesteijn M. (2017).

[bb0095] Morse J.M., Black C., Oberle K., Donahue P. (1989). A prospective study to identify the fall-prone patient. Soc. Sci. Med..

[bb0100] Muehlbauer T., Granacher U., Borde R., Hortobágyi T. (2018). Non-discriminant relationships between leg muscle strength, mass and gait performance in healthy young and old adults. Gerontology.

[bb0105] Queen R.M., Campbell J.C., Schmitt D. (2019). Gait analysis reveals that Total hip arthroplasty increases power production in the hip during level walking and stair climbing. Clin. Orthop. Relat. Res..

[bb0110] Rogind H., Simonsen H., Era P., Bliddal H. (2003). Comparison of Kistler 9861A force platform and Chattecx balance system® for measurement of postural sway: correlation and test-retest reliability. Scand. J. Med. Sci. Sports.

[bb0115] Tang W., Gan X., Zhanghui L., Huang Y. (2010). Feasibility of applying Chinese Morse fall assessment scale in clinical nursing in China. J. China Med. Univ..

[bb0120] Thomas E., Battaglia G., Patti A., Brusa J., Leonardi V., Palma A., Bellafiore M. (2019). Physical activity programs for balance and fall prevention in elderly. Medicine.

[bb0130] Tou N.X., Goh S.F., Harding S., Tsao M.A., Ng T.P., Wee S.-L. (2024). Effectiveness of community-based Baduanjin exercise intervention for older adults with varying frailty status: a randomized controlled trial. European Rev. Aging Phys. Activity : Off J. European Group Res. Elderly. Phys. Activity.

[bb0135] Wooten S.V., Signorile J.F., Desai S.S., Paine A.K., Mooney K. (2018). Yoga meditation (YoMed) and its effect on proprioception and balance function in elders who have fallen: a randomized control study. Complement. Ther. Med..

[bb0140] Ye J., Cheung W.M., Tsang H.W.H. (2019). The neuroscience of nonpharmacological traditional Chinese therapy (NTCT) for major depressive disorder: a systematic review and Meta-analysis. Evid. Based Complement. Alternat. Med..

[bb0145] Ye J., Simpson M.W., Liu Y., Lin W., Zhong W., Cai S., Zou L. (2020). The effects of Baduanjin qigong on postural stability, proprioception, and symptoms of patients with knee osteoarthritis: a randomized controlled trial. Front. Med..

[bb0150] Ye M., Zheng Y., Xiong Z., Ye B., Zheng G. (2022). Baduanjin exercise ameliorates motor function in patients with post-stroke cognitive impairment: a randomized controlled trial. Complement. Ther. Clin. Pract..

[bb0155] Zou L., Sasaki J.E., Wang H., Xiao Z., Fang Q., Zhang M. (2017). A systematic review and Meta-analysis of Baduanjin qigong for health benefits: randomized controlled trials. Evid. Based Complement. Alternat. Med..

[bb0160] Zou L., Pan Z., Yeung A., Talwar S., Wang C., Liu Y., Shu Y., Chen X., Thomas G.A. (2018). A review study on the beneficial effects of Baduanjin. J. Altern. Complement. Med..

